# A CRISPR mis-insertion in the *Zic3* 5′UTR inhibits *in vivo* translation and is predicted to result in formation of an mRNA stem-loop hairpin

**DOI:** 10.1242/bio.061677

**Published:** 2025-03-17

**Authors:** Helen M. Bellchambers, Maria B. Padua, Stephanie M. Ware

**Affiliations:** ^1^ Herman B Wells Center for Pediatric Research, Department of Pediatrics, Indiana University School of Medicine, Indianapolis, IN, 46202, USA; ^2^ Department of Medical and Molecular Genetics, Indiana University School of Medicine, Indianapolis, IN, 46202, USA

**Keywords:** RNA structure, Cardiac development, Transgenic mice, Translation control, CRISPR, Gene expression

## Abstract

*Zic3* loss of function is associated with a range of congenital defects, including heterotaxy and isolated heart defects in humans, as well as neural tube defects, situs anomalies, and tail kinks in model organisms. Here, we describe a novel *Zic3^ins5V^* mouse line generated due to a mis-insertion during the CRISPR genome editing process, which altered the *Zic3* 5′UTR structure. Mice with this insertion developed similar phenotypes to *Zic3^LacZ^* null mice, including heterotaxy, isolated heart defects, neural tube defects and tail kinks. Surprisingly, gene expression analysis revealed that the novel *Zic3^ins5V^* line displays higher levels of *Zic3* mRNA, but western blot analysis confirmed that levels of ZIC3 were greatly reduced *in vivo*. RNAfold, an RNA secondary structure prediction tool, showed that this mis-insertion may cause the formation of a large stem-loop hairpin incorporating some of the 5′UTR and first exon of *Zic3*, and the insertion of similar hairpins in a cell-based assay caused the loss of ZIC3 expression. Thus, this mouse line displays a loss of ZIC3 protein consistent with the inhibitory effects of 5′UTR stem-loop hairpin structures.

## Teaser

**Summary:** A CRISPR mouse line with a *Zic3* 5′UTR insertion predicted to result in a stem-loop hairpin, demonstrates increased *Zic3* mRNA, decreased ZIC3 protein, and loss of function phenotypes.

## INTRODUCTION

*Zic3* is an X-linked zinc finger transcription factor with a range of functions in early development (Bellchambers and Ware, 2018). In humans, *ZIC3* variants are associated with X-linked heterotaxy (Ware et al., 2004), a syndrome caused by disruption of the establishment of left-right patterning and characterized by organ laterality defects including abnormal heart looping. Mouse, frog and zebrafish models confirmed *Zic3* is critical for the establishment of left-right patterning and indicated that loss of *Zic3* function can also cause other issues such as gastrulation defects, neural tube defects (NTDs), tail kinks and isolated heart defects (Cast et al., 2012; Haaning et al., 2013; Purandare et al., 2002; Ware et al., 2006b). We have recently linked both the heterotaxy related defects and the NTDs observed in *Zic3* null mice to abnormal planar cell polarity signaling (Bellchambers and Ware, 2021), but *Zic3* is also known to regulate other signaling pathways such as Nodal (Ware et al., 2006a), canonical Wnt (Bellchambers et al., 2021; Fujimi et al., 2012) and Hedgehog (Quinn et al., 2012) signaling pathways.

RNA secondary structures (i.e. stem-loop hairpins, pseudoknots and multibranch loops) can modulate protein synthesis through different mechanisms such as altering RNA synthesis, splicing, and translation (Georgakopoulos-Soares et al., 2022). The effect of these secondary structures can be difficult to predict as the outcome can vary based on location or stability of the structure. For example, stem-loop hairpins with a thermal stability of −50 kcal/mol can repress translation when located in the 5′UTR in close proximity to the start codon (Babendure et al., 2006; Kozak, 1986) by stalling progression of the translational machinery (Wang et al., 2022). In contrast, stem-loop hairpins in the coding sequence and/or 3'UTR can increase the stability of mRNA transcripts therefore increasing the amount of protein produced (Mauger et al., 2019). Given the critical role of mRNA secondary structures in translation, several studies have suggested that hairpins can be introduced into the 5′UTR to fine tune the rate of protein synthesis (Wang and Simmel, 2022; Weenink et al., 2018). However, these studies have generally been performed in prokaryotes or mammalian cell lines due to technical challenges of introducing such structures into higher organisms.

Here, we characterized a mouse line created by a mis-insertion in the 5′UTR of *Zic3* during the CRISPR genome editing process. This CRISPR process inserted an extra 132 bp from the donor template as well as the desired V5 epitope tag at the N-terminal of the *Zic3* coding sequence. Despite the fact that the addition of the V5 tag alone did not alter *Zic3* function and that the coding sequence of *Zic3* remained intact, mice with this large insertion displayed similar phenotypes to *Zic3^LacZ^* null mice but at a lower rate. In particular, *Zic3^LacZ^* null mice displayed a 100% penetrant tail kink phenotype, which occurred at a lower rate in mice with the insertion, suggesting this mouse line was a hypomorphic *Zic3* allele. *In vivo*, we determined that this insertion induced the upregulation of *Zic3* mRNA while levels of ZIC3 protein were strongly reduced*.* An mRNA secondary structure prediction algorithm indicated that the insertion is highly likely to cause formation of a stem-loop hairpin structure via interaction of the donor template with part of the 5′UTR and first exon of *Zic3*. The introduction of similar stem-loop hairpins into *Zic3* plasmids produced a reduction in protein levels *in vitro*. As such, this unique mouse line displays loss of function phenotypes and loss of ZIC3 protein consistent with the inhibitory effects of 5′UTR stem-loop hairpin structures.

## RESULTS

### Generation of the *Zic3^ins5V^* allele

**Fig. 1.**
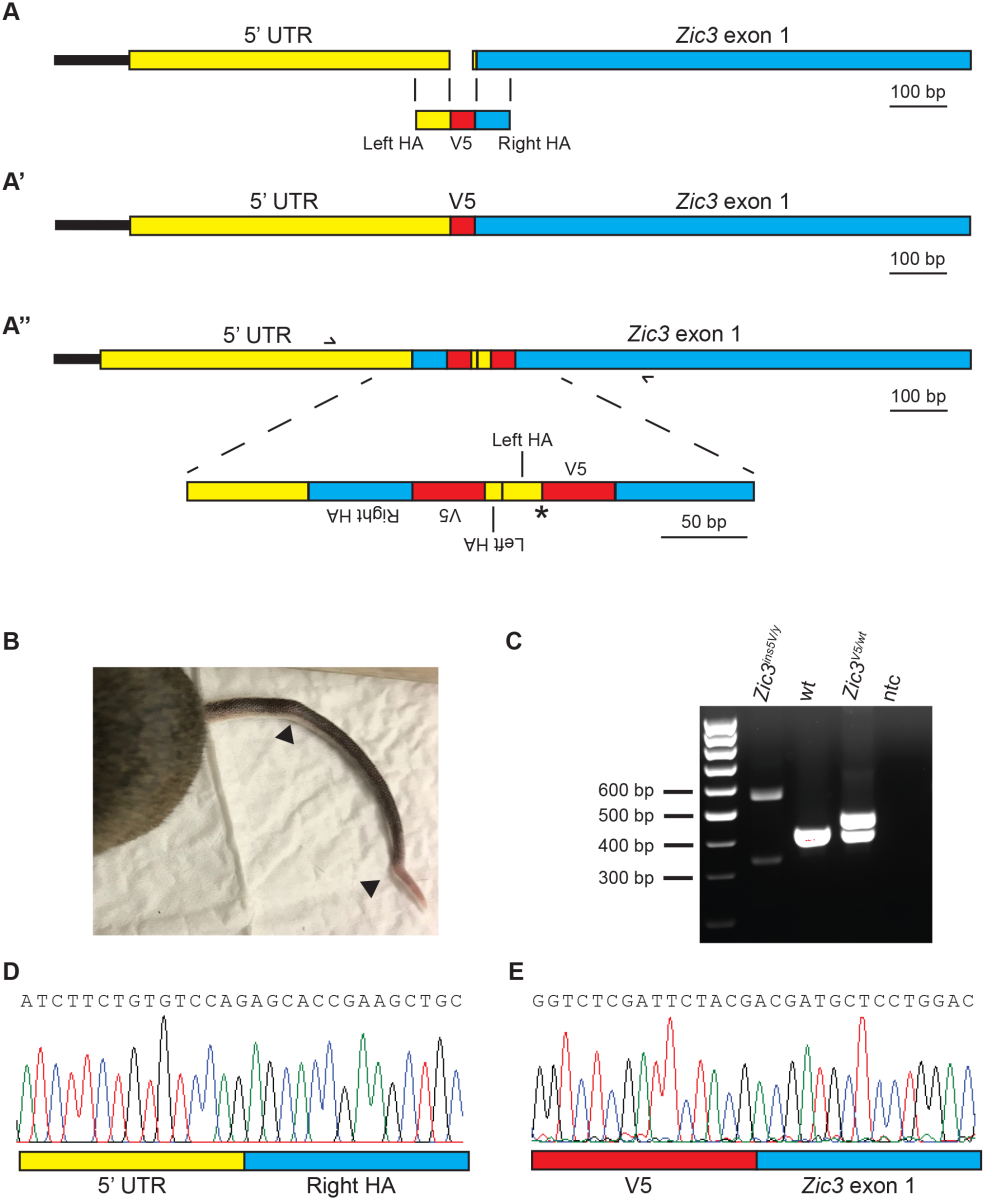
**Generation and analysis of the *Zic3^V5^* and *Zic3^ins5V^* mouse lines.** (A) Schematic illustrating the site of CRISPR cleavage position relative to *Zic3* and the homology between the donor template and genomic region, (A′) the desired V5 epitope tagged *Zic3* mouse line (*Zic3^V5^*) and (A″) the arrangement of the insertion in the *Zic3^ins5V^* mouse line. In A″, primers are shown as black half arrows and asterisk denotes the location of Kozak sequence. (B) The tail of the first *Zic3^ins5V^* mouse and tail kinks are indicated with arrowheads. (C) PCR electrophoretogram displaying the amplicon produced by the insertion in *Zic3^ins5V^* mouse using primers shown in A″. Note the amplicon is larger than the amplicons produced by either wild-type or *Zic3^V5^*^/wt^ mice, indicating an undesired large insertion occurred. (D,E) Partial Sanger sequencing chromatogram of the PCR amplicons shown in C of a *Zic3^ins5V/y^* mouse showing that (D) the right homology arm has been inserted into the 5′UTR and (E) the V5 tag was inserted within *Zic3* exon 1. HA, homology arm; wt, wild-type; ntc, no template control.

To investigate the function of *Zic3* during early development, we attempted to generate a *Zic3^V5^* mouse line in which a V5 epitope tag was inserted at the N-terminus of *Zic3*. CRISPR reagents were designed to cut 7-12 bp before the translational start of *Zic3* and insert the V5 tag via homologous direct repair with a donor template (Fig. 1A,A′). The resulting mice were deep sequenced to identify founders with high levels of incorporation of the desired V5 tag, which were then crossed with wild-type mice to confirm germline transmission of the insertion. Genotyping of the subsequent pups indicated all mice produced some offspring with the correct insertion of the V5 tag. However, one pup did not produce any bands in the genotyping assay (Fig. S1Ai-ii) and at the time of weaning a mild tail kink was detected (Fig. 1B), which is a phenotype previously observed in *Zic3* null animals (Ahmed et al., 2013; Jiang et al., 2013; Purandare et al., 2002), although the tail kinks developed by *Zic3* null mice are more severe.

One possibility was that the CRISPR process produced a large deletion disrupting sites of primer binding. However, PCR with primers targeting the regions of *Zic3* 5′UTR and exon 1 adjacent to the CRISPR cut site produced identical fragments to wild-type, indicating these regions remained intact (Fig. S1Bi-ii). To characterize this mouse, primers were designed within the regions of the *Zic3* 5′UTR and exon 1 that were confirmed to be present, but that would amplify across the region targeted by CRISPR (Fig. S1C). The resulting PCR fragment was ∼110 bp larger than expected if the V5 tag had incorporated correctly (Fig. 1C), suggesting that the CRISPR process had inserted a larger DNA fragment than the desired epitope tag. The resulting PCR fragment was also less intense than the wild-type control fragment (Fig. 1C), suggesting PCR was inhibited by the insertion. Sanger sequencing of the PCR fragment showed that the V5 epitope tag had been incorporated at the start of *Zic3*, but additional regions were also incorporated; specifically 23 bp of the left homology arm remained adjacent to the V5 epitope tag and a second copy of the majority of the donor template (specifically, the entire right homology arm, the V5 epitope tag and 10 bp of the left homology arm) had also been inserted in the reverse orientation, upstream of the left homology arm/V5 insertion (Fig. 1A″,D,E). As such, the allele was named *Zic3^ins5V^*. Further PCR with primers targeting exon 1, exon 2 and exon 3 of *Zic3* produced identical fragments to wild-type, and Sanger sequencing indicated the coding sequence of *Zic3* remained intact (data not shown).

### *Zic3^ins5V^* mice display reduced viability and a tail kink phenotype

The tail kink phenotype suggests that *Zic3* function may be impaired in these mice. Since the loss of *Zic3* results in significant lethality, we measured the viability of *Zic3^ins5V^* mice. Heterozygous *Zic3^ins5V/wt^* females were crossed with wild-type males and offspring genotyped at 2 weeks of age. The ratios of the resulting progenies differed significantly from the expected Mendelian ratios (*P*=0.011), indicating partial lethality of those with the allele (Table 1), suggesting *Zic3* function is affected in this line. To determine when the lethality is occurring, heterozygous *Zic3^ins5V/wt^* females were crossed with wild-type males and the resulting embryos were collected at 13.5-14.5 dpc. There was no significant deviation from the expected Mendelian ratios at this time point, indicating no loss of viability at this stage (Table 1).

**Table 1.** *Zic3^ins5V^* mice display reduced viability

Age	wt female	wt male	*Zic3^ins5V^* ^/*wt*^	*Zic3^ins5V/y^*	total	*P*-value
Adult	90	75	102	62	329	0.011
13.5-14.5 dpc	15	15	12	17	58	0.834

*P***-**value calculated via Chi-squared analysis. wt, wild-type.

Previous studies of several *Zic3* mouse alleles indicate loss of *Zic3* function causes a 100% penetrant tail kink phenotype (Ahmed et al., 2013; Jiang et al., 2013; Purandare et al., 2002). Hence, the *Zic3^ins5V/y^* males produced by this cross were assessed for this phenotype. Tail kinks were detected in 60% (37/62) of *Zic3^ins5V/y^* males, indicating this phenotype was present but only partially penetrant*.* To determine if the rate of phenotype was affected by the background strain, we reassessed the tail kink phenotype after at least five generations of backcross to the 129B6/Smwa strain, on which the *Zic3-LacZ* line is maintained and displays a 100% penetrant tail kink phenotype. At that point the *Zic3^ins5V/y^* line was 96.9% identical to the 129B6/Smwa strain and is therefore considered incipient congenic. Only 80% (12/15) of *Zic3^ins5V/y^* males had tail kinks, confirming the phenotype remains partially penetrant regardless of the background indicating that the insertion has created a hypomorphic allele.

### *Zic3^ins5V^* mice have neural tube and heart defects

The loss of *Zic3* has previously been associated with several embryonic phenotypes in mice, including NTDs, heterotaxy and isolated heart defects (Bellchambers and Ware, 2021; Haaning et al., 2013; Purandare et al., 2002; Ware et al., 2006a). To further assess whether *Zic3* function was altered in this line, we collected 28 homozygous or hemizygous *Zic3^LacZ^* null embryos and 59 homozygous or hemizygous *Zic3^ins5V^* embryos at 13.5-14.5 dpc which were examined for any of the previously observed phenotypes.

**Fig. 2.**
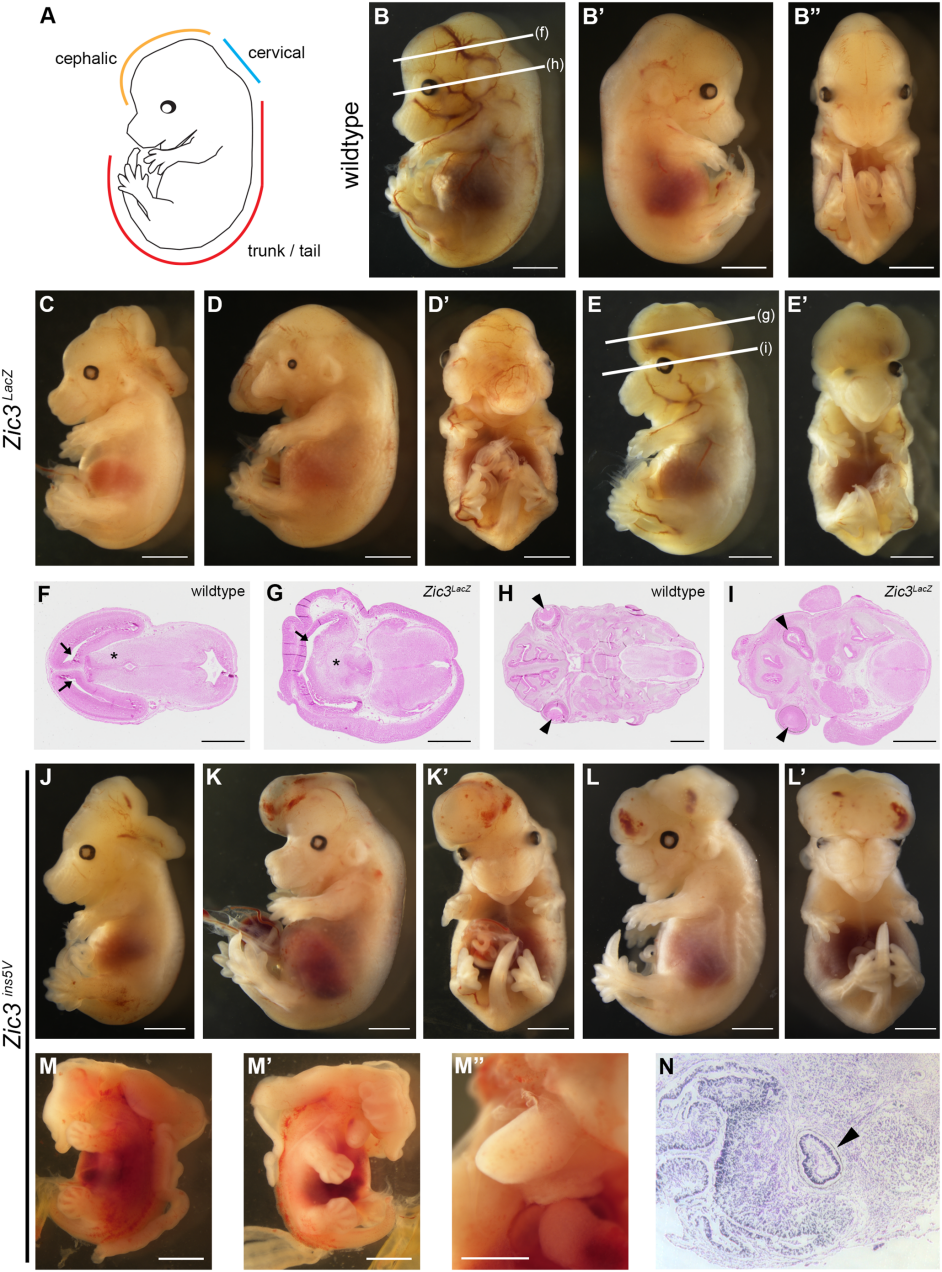
**Neural tube defects observed in *Zic3^LacZ^ and Zic3^ins5V^* embryos.** (A) Lateral view illustration of a 14.5 dpc embryo showing regions of the neural tube (adapted from Bellchambers et al., 2021). (B-B′) Lateral and (B″) frontal views of a wild-type embryo. White lines in B indicate regions sectioned in F and H. (C) Lateral view of *Zic3^LacZ/LacZ^* null embryo with a neural tube defect in the cervical region. (D) Lateral and (D′) frontal views of a *Zic3^LacZ/y^* null embryo with a neural tube defect in the cephalic region. (E) Lateral and frontal (E′) views of a *Zic3^LacZ/LacZ^* null embryo with midline facial defects. White lines in E indicate regions sectioned in G and I. (F) Section of wild-type embryo forebrain with two separate lateral/telencephalic ventricles and the third ventricle at the midline of the diencephalon. (G) Section of *Zic3^LacZ/LacZ^* embryo forebrain displaying a single fused ventricle and lack of the third ventricle in the diencephalon. (H) Section of wild-type embryo head with two eyes on surface of the embryo. (I) Section of *Zic3^LacZ/LacZ^* embryo head indicating one of the eyes is dysmorphic and located abnormally close to the midline. (J) Lateral view of *Zic3^ins5V^*^/*ins5V*^ embryo with a neural tube defect in the cervical region. (K) Lateral and (K′) frontal views of a *Zic3^ins5V/y^* embryo with an encephalocele in the cephalic region. (L) Lateral and frontal (L′) views of a *Zic3^ins5V/y^* embryo with a neural tube defect in the cephalic region. (M-M′) Lateral and (M″) frontal views of a *Zic3^ins5V/y^* embryo with craniorachischisis and several facial defects. (N) Transverse section of embryo shown in (M) taken from the head at the level of the eye and stained with Hematoxylin and Eosin. Arrows point to lateral ventricles, arrowheads indicate eyes, and asterisk (*) denotes diencephalon. (B-E′, J-L′ and M-M′) scale bar: 2 mm; (F-I and M″) scale bar: 1 mm.

Seven of *Zic3^LacZ^* null embryos (25%) exhibited NTDs (Fig. 2A-E), with four displaying a NTD restricted to the cervical region, which is the region most commonly affected in *Zic3* null embryos (Bellchambers and Ware, 2021), and two embryos displaying NTDs in the cephalic region. In addition, one embryo presented with a NTD affecting both the cephalic and cervical regions, as well as midline facial defects, including a fused upper lip and a hypoplastic mid-face. These defects are most typically observed in holoprosencephaly mouse models (Hong and Krauss, 2018), therefore the head of this embryo was examined histologically. Brain sections revealed a fusion of lateral/telencephalic ventricles and an absence of the third ventricle within the diencephalon (Fig. 2F,G), which are forebrain defects that occur as part of the holoprosencephaly spectrum. In addition, one eye was positioned abnormally close to the midline (Fig. 2H,I).

Four of the *Zic3^ins5V^* embryos (6.8%) presented NTDs (Fig. 2J-M). Of these, one embryo had the NTD restricted to the cervical region as is typically observed in the *Zic3* null embryos (Fig. 2J). Two embryos displayed NTDs in the cephalic region (Fig. 2K-K′,L-L′). Another embryo had craniorachischisis with facial dysmorphia including a hypoplastic mid-face with a proboscis like structure and cyclopia, which are midline defects typically associated with holoprosencephaly (Fig. 2M-M″,N). Notably, comparison of the rate of NTDs in the *Zic3^ins5V^* line to the *Zic3^LacZ^* null line indicated that the NTDs were occurring at a lower rate (6.8% *Zic3^ins5V^* versus 25% *Zic3* null embryos).

**Fig. 3.**
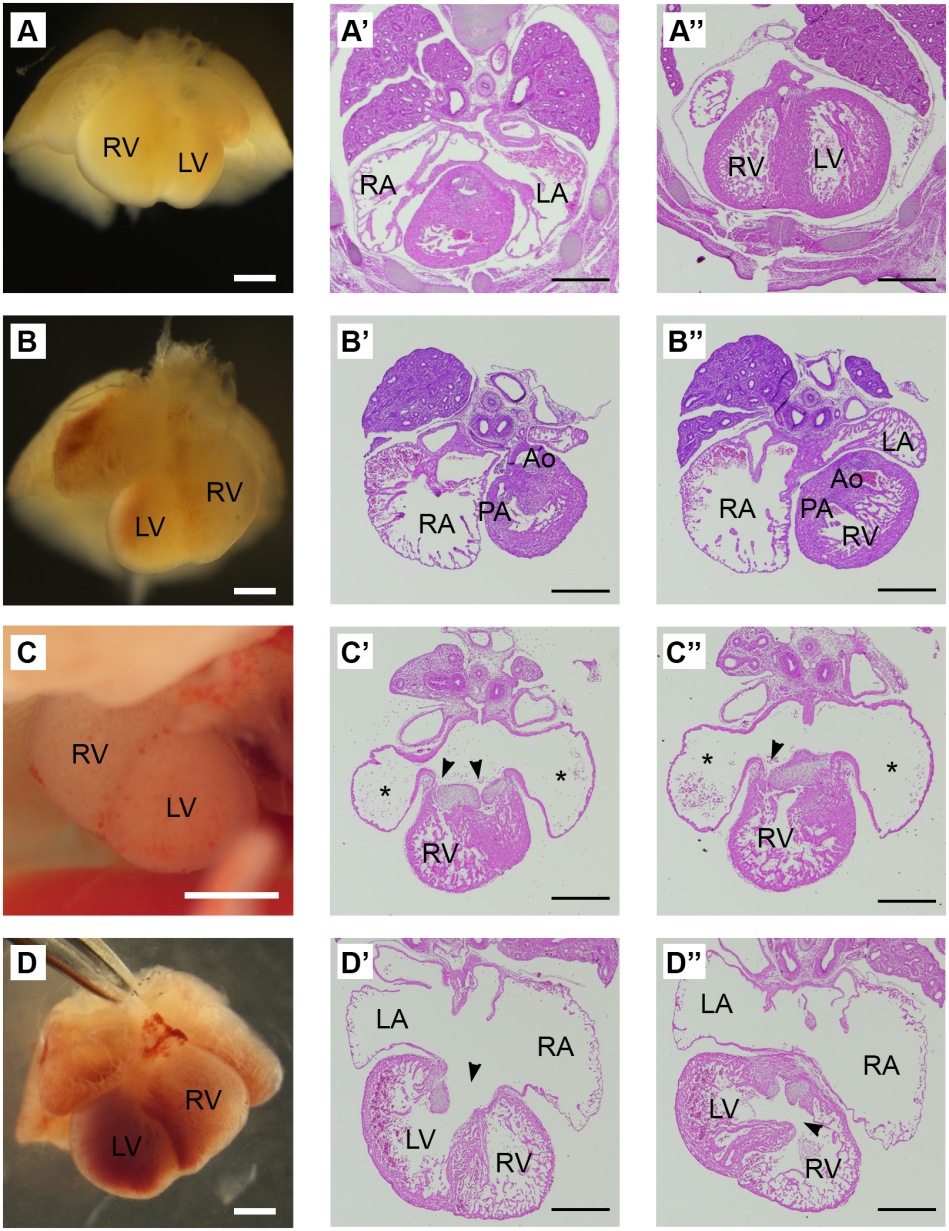
***Zic3^ins5V^* embryos exhibit heart defects.** (A-D) Gross embryonic hearts at 13.5-14.5 dpc and (A′-D″) transverse sections stained with Hematoxylin and Eosin. (A-A″) Wild-type heart with proper orientation of ventricles. (B-D) Heart looping defects found in *Zic3^ins5V^* embryos, including (B) dextrocardia, (C) incomplete looping and (D) dextrocardia with incomplete looping. In addition to looping defects, *Zic3^ins5V^* heart sections also revealed (B′-B″) a double outlet right ventricle (DORV) defect. (C′-C″) Atrial isomerism with an atrioventricular canal defect. (D′-D″) Atrioventricular canal defect and thin wall. Arrowheads point to atrioventricular canal defects and asterisks (*) denote atrial isomerism. RA, right atrium; LA, left atrium; RV, right ventricle; LV, left ventricle; PA, pulmonary artery; Ao, aorta. Scale bars: 500 µm.

Hearts were examined both grossly and histologically (Fig. 3A-D,A′-D′,A″-D″). *Zic3^ins5V^* hearts showed gross defects consistent with those previously observed in *Zic3* null hearts, including sinistral looping (4/59 hearts; Fig. 3B,D) and incomplete looping (7/59 hearts; Fig. 3C,D). As some hearts showed multiple defects, a total of 15.3% (9/59) *Zic3^ins5V^* hearts had a gross anatomic defect. Heart sections also showed additional defects including, double outlet right ventricles (11/59 hearts; Fig. 3B′-B″), atrial isomerism (1/59 hearts; Fig. 3C′-C″), ventricular septal defects (13/59; Fig. 3D″), atrioventricular canal defects (2/59 hearts; Fig. 3C′-C″,D′-D″) and thin walls (1/59 hearts; Fig. 3D′-D″). All these defects have previously been associated with loss of *Zic3* (Bellchambers and Ware, 2021; Haaning et al., 2013; Purandare et al., 2002; Ware et al., 2006a). To determine whether heart defects occurred in the context of other laterality defects, lung lobation and position of the stomach were also observed for the 14.5 dpc embryos. Four out of 56 embryos (7.1%) were found to have abnormal lung lobation (either right isomerism, left isomerism, or reversal of the lung lobes; data not shown) in addition to the heart defects. Together, these results suggest that the *Zic3^ins5V^* line displays the full spectrum of isolated heart defects and heterotaxy that has previously been associated with loss of *Zic3*.

To compare the rate of defects, the hearts, lungs and stomachs of the *Zic3^LacZ^* null hemizygous or homozygous embryos were also examined. Ten out of 28 (35.7%) *Zic3^LacZ^* hearts exhibited sinistral and/or incomplete looping (data not shown). For six of these embryos (21.4%), the heart defect occurred in conjunction with abnormal lung lobation or a right sided stomach, consistent with heterotaxy (data not shown). Thus, similar to the NTDs, the overall rate of heart defects (15.3% *Zic3^ins5V^* versus 35.7% *Zic3^LacZ^* null embryos) and heterotaxy (7.1% *Zic3^ins5V^* versus 21.4% *Zic3^LacZ^*) was lower in *Zic3^ins5V^* embryos than in *Zic3* null embryos.

### The V5 epitope tag does not disrupt *Zic3* activity

The phenotypes observed in the *Zic3^ins5V^* embryos/mice (tail kink, NTDs and heart defects) suggest that *Zic3* function is altered by the insertion. Although the V5 tag was inserted in frame with *Zic3* and thus *Zic3^ins5V^* embryos/mice were expected to still produce full length ZIC3 protein, it is possible the addition of the V5 epitope tag to the N-terminus altered *Zic3* activity. However, if the phenotype arose from the addition of the V5 epitope tag, then the *Zic3^V5^* mice should also show similar phenotypes. To explore this possibility, 37 *Zic3^V5^* embryos were collected at 14.5-16.5 dpc for NTDs and/or gross heart defects assessment. None of the *Zic3^V5^* embryos examined showed any heart defects, heterotaxy or cephalic/cervical NTD. Only one embryo displayed spina bifida (data not shown), indicating that the addition of the V5 epitope tag was not interfering with the function of ZIC3.

### Increased expression of *Zic3* mRNA but reduced levels of ZIC3 protein in *Zic3^ins5V^* embryos

**Fig. 4.**
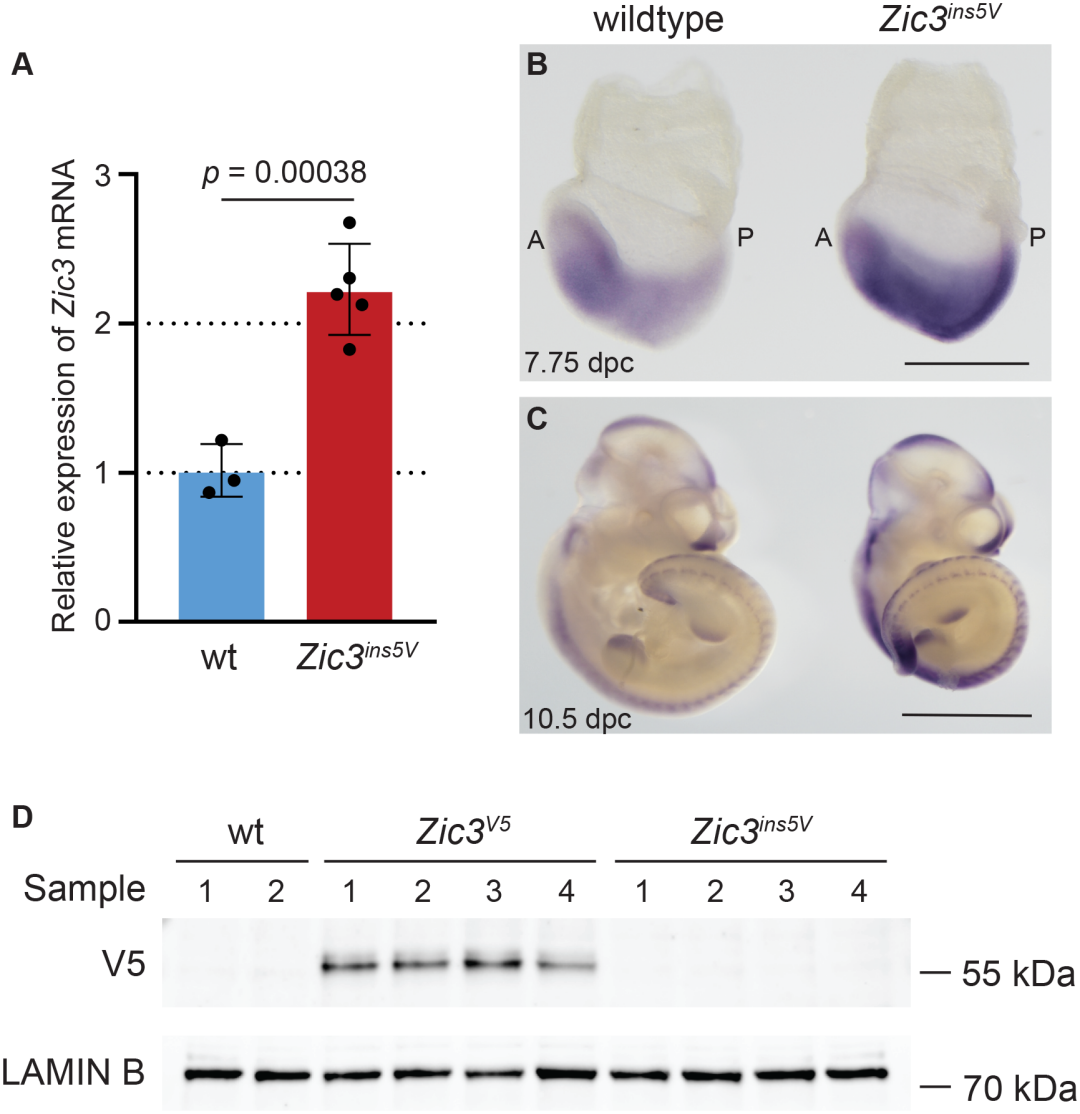
***Zic3* is overexpressed and undertranslated in *Zic3^ins5V^* embryos.** (A) Relative expression levels of *Zic3* in wild-type (*n*=3; wt, blue bar) and *Zic3^ins5V^* (*n*=5; red bar) embryos at the headfold stage (7.75 dpc) were assessed using Taqman probes for qPCR. *Tbp* was used for data normalization. Bars display geometric means and error bars represent geometric standard deviation (SD). Dots represent individual samples. (B) Whole-mount *in situ* hybridization using a riboprobe for *Zic3* on 7.75 dpc wild-type (*n*=13) and *Zic3^ins5V^* (*n*=9) embryos; scale bar: 250 µm. (C) Whole-mount *in situ* hybridization using a riboprobe for *Zic3* on 10.5 dpc wild-type (*n*=7) and *Zic3^ins5V^* (*n*=6) embryos; scale bar: 2000 µm. Purple staining in (B) headfold and primitive streak and (C) somites, limb buds and brain. A and P indicate anterior and posterior of embryos, respectively. (D) Western blot images for V5 (ZIC3) from nuclear enriched lysates of wild-type (*n*=2), *Zic3^V5^* (*n*=4) and *Zic3^ins5V^* (*n*=4) 10.5 dpc embryos. LAMIN B served as nuclear loading control. Full blots are shown in Fig. S3.

Given the 5′UTR has critical roles in regulating both transcription and translation, it is possible that the insertion disrupts the level of either *Zic3* mRNA or protein. To assess for altered transcription, we measured the level of *Zic3* mRNA in 7.75 dpc embryos via qPCR. Surprisingly, *Zic3^ins5V^* embryos had around a 2.2-fold increase in the level of *Zic3* mRNA when compared to wild-type embryos (*P*=0.00038) (Fig. 4A, Fig. S2). To determine whether *Zic3* overexpression is due to increased levels of endogenous expression or ectopic expression, we performed whole-mount *in situ* hybridization at 7.75 dpc and 10.5 dpc. The expression pattern of *Zic3* was consistent with the previously reported expression in the headfold, mesendoderm and primitive streak at 7.75 dpc as well as in the somites, limb-buds, and the part of the brain at 10.5 dpc in both the wild-type and *Zic3^ins5V^* embryos (Fig. 4B,C). Fig. 4 shows representative examples of the stained embryos, illustrating the reproducibly increased intensity observed in the *Zic3^ins5V^* embryos compared wild-type embryos. While the staining was more intense, the overall pattern remained the same between wild-type and *Zic3^ins5V^* embryos. Thus, the increase in expression was not due to ectopic expression, but increased levels of endogenous mRNA expression.

The increased levels of *Zic3* mRNA is intriguing as *Zic3* overexpression has been associated with heart defects in mouse (Zhu et al., 2007), zebrafish (Paulussen et al., 2016) and *Xenopus* (Kitaguchi et al., 2000). However, changes in transcription do not always correlate with changes in translation. As such, the levels of ZIC3 protein were assessed in embryos by western blot analysis. It has been historically challenging to assess ZIC3 protein levels *in vivo* due to the conservation between different ZIC family members resulting in non-isoform specific ZIC antibodies. Therefore, the analysis was carried out using a V5 antibody, with comparison to *Zic3^V5^*. Wild-type embryos were also included to confirm the specificity of the antibody. *Zic3^V5^* 10.5 dpc embryo lysates produced a clear immunoreactive band consistent with the molecular weight (MW) of ZIC3 (predicted MW ∼52 kDa including the V5 epitope tag) that was not present in the wild-type samples (Fig. 4D, Fig. S3A,B). However, no band corresponding to ZIC3 was detected in *Zic3^ins5V^* lysates, indicating that at the protein level ZIC3 is strongly reduced in this line.

### The *Zic3^ins5V^* insertion is predicted to form a large RNA hairpin that inhibits protein production *in vitro*

**Fig. 5.**
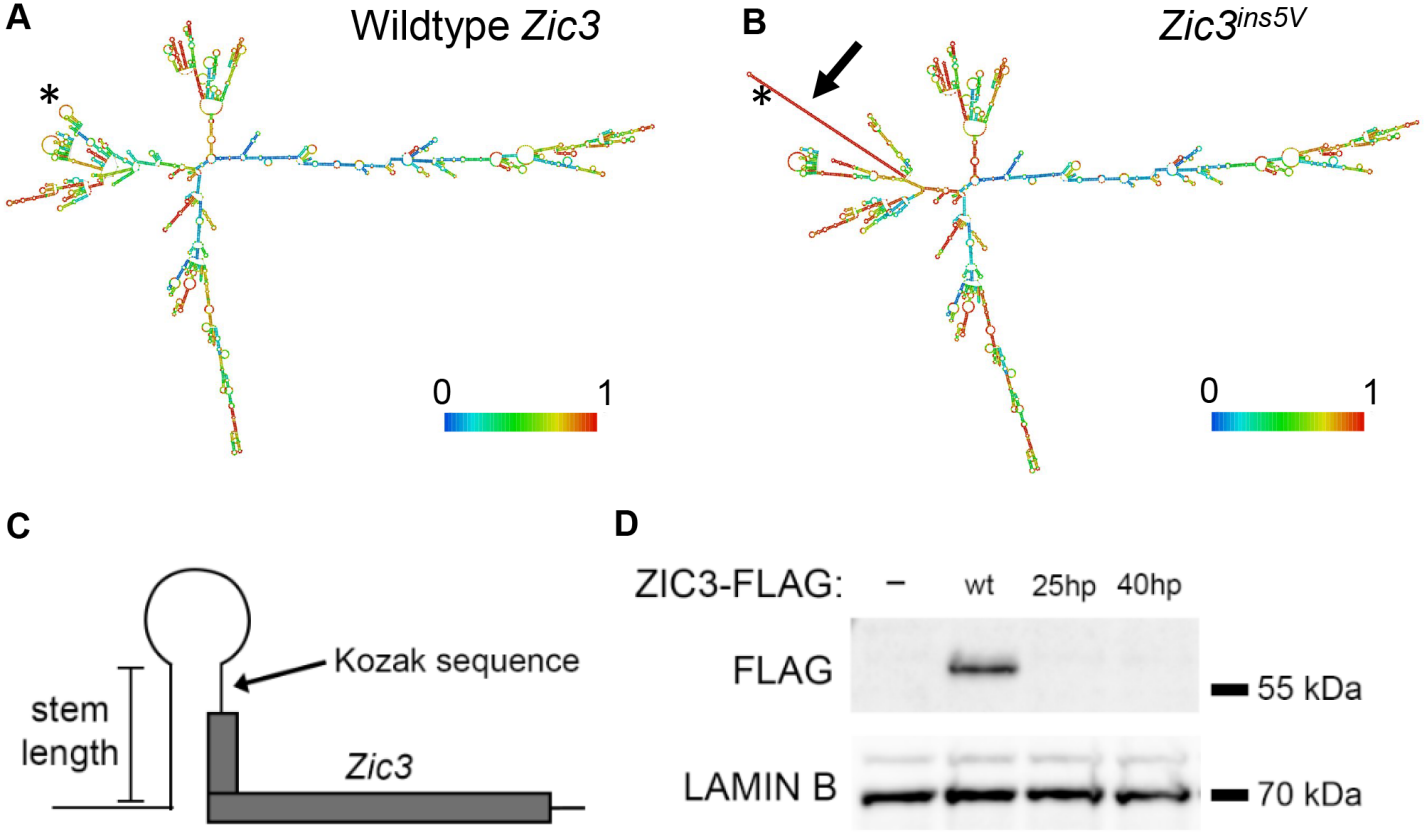
***Zic3^ins5V^* mRNA is predicted to contain a stem-loop hairpin structure.** Predicted secondary structure of (A) *Zic3^w^*^t^ and (B) *Zic3^ins5V^* via RNAfold. Scale indicates the base-pair probability, with red bases having the highest probability. The novel secondary structure is indicated by the thick arrow and the asterisk denotes the start codon location. (C) Schematic of hairpin generated in *Zic3* plasmids. (D) Representative western blot image for FLAG (ZIC3) from NIH3T3 nuclear lysates. LAMIN B served as nuclear loading control. For each plasmid, *n*=3 independent transfections and western blots. -, untransfected; wt, wild-type mouse *Zic3* (FLAG tagged) plasmid; 25 hp and 40 hp are mouse *Zic3* plasmids with 25 bp and 40 bp stem hairpins, respectively. Full blots shown in Fig. S3.

The insertion incorporated into the 5′UTR in *Zic3^ins5V^* included homology to portions of *Zic3* 5′UTR and exon 1 creating a repetitive region of DNA. We thus hypothesized the repetitive nature of the region might cause the formation of a secondary structure. To examine potential changes to the *Zic3* secondary structure, RNAfold (Gruber et al., 2008; Lorenz et al., 2011) was used to predict the secondary structure of wild-type *Zic3* mRNA and *Zic3^ins5V^* mRNA. When the secondary structures were compared, it was found that the insertion was predicted to cause the formation of a novel large hairpin structure, which included the *Zic*3 start codon (Fig. 5A,B, Fig. S4).

To determine whether the novel hairpin in the *Zic3* 5′UTR could affect ZIC3 protein levels, hairpins were introduced into a similar region of a mouse *Zic3* plasmid containing a C-terminal myc-FLAG tag. We initially attempted to introduce a hairpin with a 117 bp stem to match the *in vivo* insertion; however, the plasmid was very unstable, and we were consequently unable to purify enough DNA for further experiments. Instead, two smaller hairpins were assessed, one with a 25 bp stem and the other with a 40 bp stem, both of which incorporated the Kozak sequence upstream of ZIC3 (Fig. 5C). The wild-type myc-FLAG tagged mouse *Zic3* plasmid produced an immunoreactive band consistent with the MW of ZIC3 (predicted MW ∼54 kDa including the myc-FLAG epitope tag) when transfected into NIH3T3 cells, whereas the untransfected cells did not yield any immunoreactive band (Fig. 5D, Fig. S3C,D). In contrast, neither the 25 bp nor the 40 bp hairpin plasmids produced detectable immunoreactive band when transfected into NIH3T3 cells, indicating that 5′UTR hairpins have a strong inhibitory effect on ZIC3 protein production.

## DISCUSSION

Our study characterizes a novel mouse model where an unexpected integration of a CRISPR repair template in the 5′UTR of *Zic3* is predicted to result in a 5′UTR stem-loop hairpin. The repetitive nature of stem-loop hairpins makes these structures challenging to engineer *in vivo* via traditional methods; hence, studies on mRNA stem-loop hairpins have historically relied on bacteria, mammalian cells or yeast (Babendure et al., 2006; Espah Borujeni et al., 2017; Kozak, 1986; Ringnér and Krogh, 2005; Wang and Simmel, 2022). Less in known about the consequences of introducing stem-loop hairpins within higher eukaryotic *in vivo* models. The tail kinks, NTDs and heart defects observed in this line suggest the insertion generated a loss of function allele of *Zic3*. This insertion increased *Zic3* mRNA levels, while reducing ZIC3 protein levels, and thus represents an example of changes to transcription not correlating with changes in translation.

There have been other cases of altered mRNA secondary structure producing differing effects on the transcript and protein levels. For example, expansion of a trinucleotide repeat within the *FMR1* 5′UTR can cause a loss of function resulting in Fragile X-syndrome (Ajjugal et al., 2021; Peprah, 2012). The extent of the loss of function depends on the length of the expansion, but smaller expansions (sometimes called premutations) have been shown to cause a partial loss of protein whereas transcript levels were unaltered or elevated (Peprah et al., 2010; Primerano et al., 2002; Tassone et al., 2007). It has been hypothesized that the expansion of repetitive sequences causes the formation of a strong secondary structure which slows or stalls translational scanning, though there is some debate whether this is a stem-loop hairpin or a quadruplex structure (Ajjugal et al., 2021; Zumwalt et al., 2007).

Stem-loop hairpins were originally hypothesized to repress translation when located in the 5′UTR either by blocking binding of certain translational machinery proteins or stalling progression of the ribosome. A recent elegant study that monitored the movement of different ribosomal subunits using single molecular fluorescence spectroscopy indicated that the presence of a hairpin incorporating the start codon caused the 43S pre-initiation complex to move backwards (3′ to 5′ direction) upon reaching the start codon and impaired joining of the 60S subunit, thus inhibiting translation (Wang et al., 2022). Thus, it is possible that a similar stalling of ribosome progression could cause the loss of ZIC3 protein in the *Zic3^ins5V^* mouse line. One alternative explanation for the reduction in ZIC3 protein levels in this mouse line is that the 5′UTR insertion disrupted an enhancer or promoter region. However, if that were the case, we would have expected either alteration of the *Zic3* expression pattern, or reduction of the overall level of *Zic3* mRNA. We confirmed that the expression pattern was unaltered, indicating that the loss of protein and observed phenotypes were not due to a disrupted enhancer. In fact, the overall level of *Zic3* mRNA was increased, which could be indicative of a feedback loop reacting to the loss of *Zic3* function, however, that remains to be tested.

Next generation sequencing approaches as well as Sanger sequencing are commonly used to evaluate the outcomes of CRISPR mutagenesis (Anderson et al., 2018; Cromer et al., 2022; Shah et al., 2015). The workflows of these technologies require PCR, which is known to be strongly inhibited by secondary structures such as stem-loop hairpins (Nelms and Labosky, 2011); therefore, secondary structures are difficult to detect by these methods, particularly when they are present in a heterozygous state, where the other copy of the gene (whether it was wild-type or contained a desired mutation) would be strongly favored. For example, the initial screening of the *Zic3^ins5V^* mouse line via deep sequencing failed to detect the insertion. We were only able to detect this insertion in subsequent generations because *Zic3* is on the X-chromosome and the mutation was therefore hemizygous in male offspring. As such, the existence of this mouse line suggests current screening methods are not capable of detecting certain insertions and thus highlights the need for care in assessing the outcomes of CRISPR. Newer techniques such as CRISPR-Cas9 long-read sequencing are being developed, which allows PCR-free analysis of transgenic mice (Bryant et al., 2023). However, a mouse must be euthanized to acquire enough DNA to undergo this sequencing process; as such, the developers of the technology emphasize it is not currently useful for initial screening of founder lines (Bryant et al., 2023).

In summary, this study identifies a novel *Zic3* hypomorphic mouse line resulting from an unexpected CRISPR insertion missed by typical screening processes. As this mouse line is predicted to form a stem-loop hairpin and displays both phenotypes consistent with loss of *Zic3* function as well as reduced protein production, this mouse line may potentially be useful for additional investigation of mammalian stem-loop hairpins.

## MATERIALS AND METHODS

### Mouse lines

The *Zic3^V5^* (*Zic3^em2Smwa^*; MGI:7614499) and *Zic3^ins5V^* (*Zic3^em1Smwa^*; MGI:7614497) mouse (*Mus musculus*) lines were generated and sequenced by the Genome Engineering and iPSC Center (GEiC) at Washington University, St Louis, MO, USA. Briefly, the following CRISPR guide RNA and donor template were designed to cut at the 7-12 bp before the start codon of *Zic3* and insert the V5 tag via homologous direct repair: guide RNA 5′-CCGTCCAGGAGCATCGTCANGG-3′ and 5′-CCAGGAGCATCGTCATAGGTNGG-3′; Donor repair template: 5′-TTCGCCTGCACCCTTGCTCACTTCGGCCGGATCTTCTGTGTCCAGAACACCCTACCTATGGGTAAGCCTATCCCTAACCCTCTCCTCGGTCTCGATTCTACGACGATGCTCCTGGACGGAGGCCCGCAGTTCCCTGGGTTGGGAGTGGGCAGCTTCGGTGCT-3′. The guide RNA and donor template were validated by nucleofection into N2A cells.

Knock-in mice were generated by injection of the guide RNA and donor template into hybrid C57BL6/CBA oocytes. GEiC analyzed the resulting 25 mice by targeted deep sequencing using the following primers: Forward 5′-CCA GGC AGT GTT CAA CCG CC-3′ and Reverse 5′-GAA GGG ATT CAA TCC CAT GCC-3′. Of those 25 mice, only five showed >50% of alleles containing the V5 tag in frame with no additional mutations.

These five mice were then crossed to 129B6/Smwa mice, and the resulting pups genotyped by PCR and Sanger sequencing (ACGT Inc., Wheeling, IL, USA) to confirm germline transmission and in-frame incorporation of the V5 tag. Mice were backcrossed to the 129B6/Smwa inbred strain for three generations before any assessment of phenotype or viability. The resulting mice were genotyped from ear clip DNA with the following primers:

*Zic3^V5^* mice: 5′-CTT CAG GGA TCT CCT TCG CC-3′ and 5′-TTG GGC ATC TCG TGG TGG-3′; for *Zic3^ins5V^*: 5′-AGA CTC TCG CAG CCT AGG AA-3′, 5′-ATA ACC TGA ACC CTG CGG TG-3′ and 5′-GGT AAG CCT ATC CCT AAC C-3′.

The *Zic3^LacZ^* (also called *Zic3*-*LacZ*) (Purandare et al., 2002; Ware et al., 2006b) was initially generated on a mixed C57BL6×129 background and has been maintained on the same background via brother-sister matings. As these sibling matings have occurred for greater than 20 generations, the line now exists as an inbred strain named 129B6/Smwa. The sex of the embryos and the genotype of *Zic3^LacZ^* mice/embryos was determined with the *Zic3^LacZ^* genotyping assay described elsewhere (Purandare et al., 2002; Ware et al., 2006b).

Mice were housed in the AAALAC accredited Indiana University School of Medicine Animal Facility and experiments were approved by the Institutional Animal Care and Use Committee. Authors complied with ARRIVE guidelines.

### DNA sequencing

The sequence of the insertion in the *Zic3^ins5V^* mouse was determined via a combination of PCR, cloning, and Sanger sequencing. Briefly, initial attempts to PCR the region failed, consequently eight combinations of primers were tested, of which only one produced a PCR amplicon. The size of the amplicon was determined using the ChemiDoc system and Image Lab software (Bio-Rad Laboratories, Hercules, CA, USA). The amplicon was purified and analyzed via Sanger sequencing (ACGT Inc., Wheeling, IL, USA). The sequencing trace did not cover the entire fragment (likely due to the inhibitive effects of the hairpin) but indicated that the V5 tag was present and showed that at least half of the repair template had been inserted in the reverse orientation (i.e. that a second copy of the V5 tag was present in the reverse orientation). The remainder of the sequence was determined by cutting the PCR amplicon with the restriction enzyme BseRI (R0581S, New England Biolabs, Ipswich. MA, USA), which has a recognition site with the V5 tag. The resulting fragments were blunted with the Quick Blunting kit (E1201S, New England Biolabs) and cloned into EcoRV restriction enzyme site of pSF-CMV-NH2-3xFLAG (OGS620, MilliporeSigma, Burlington, MA, USA). Colony PCR served to identify clones with the desired insert, which were purified and further analyzed via Sanger sequencing. Additional PCR reactions were performed to confirm that the *Zic3* exons and part of the 5′UTR were intact. Primers used for PCR and sequencing are listed in Table S1.

### RNA secondary structure prediction

The secondary structure of wild-type and mutant *Zic3* mRNA was predicted using RNAfold (RRID:SCR_024427; ViennaRNA Package Version 2.5.1; http://rna.tbi.univie.ac.at/cgi-bin/RNAWebSuite/RNAfold.cgi) with the default settings.

### Expression constructs

The wild-type mouse *Zic3* plasmid containing a C-terminal myc-FLAG tag was obtained from OriGene (MR223858; OriGene Technologies, Rockville, MD, USA). To generate the stem-loop hairpin plasmids, PCR fragments were amplified from the wild-type plasmid and inserted adjacent to the coding sequence using KpnI and SalI restriction enzyme sites.

### Cell culture

NIH3T3 cells were obtained from Dr. Anthony Firulli of Indiana University. Cells were cultured in high glucose Dulbecco's Modification of Eagle's Medium (DMEM) (with L-Glut and Na+ pyruvate) (HyClone, Cytiva, MA, USA) supplemented with 10% (v/v) calf bovine serum (ATCC, Manassas, VA, USA) at 37°C and 5% CO_2_ in a Forma Series II 3110 water-jacketed CO_2_ incubator. 1.2×10^6^ of NIH3T3 cells were transfected using Lipofectamine 2000 as per the manufacturer's instructions with 4 μg of plasmid. The cell line has not recently been authenticated or tested for contamination.

### Protein extraction and western blotting

Whole wild-type, *Zic3^ins5V^* and *Zic3^V5^* 10.5 dpc embryos were homogenized in 200 µl of cytoplasmic extraction reagent I from the NE-PER nuclear and cytoplasmic extraction reagents kit (Thermo Fisher Scientific) containing halt protease and phosphatase inhibitor cocktail (Thermo Fisher Scientific). Homogenized embryos and NIH3T3 cells were processed according to the manufacturer's instructions to produce nuclear and cytoplasmic enriched lysates. Concentration of the nuclear lysates was determined using the pierce BCA protein assay kit (Thermo Fisher Scientific). 50 mM DTT and 1x Laemmli buffer (Bio-Rad) were added to the lysates which were incubated for 9 min at 70°C. A total of 5 µg of protein from each nuclear lysate was loaded on a 7.5% TGX PAGE gel (Bio-Rad), which was run at 150 V before being transferred to a PVDF membrane (MilliporeSigma). Proteins were detected with the following antibodies: V5 (1:2000, #13202, RRID: AB_2687461; Cell Signaling Technology, Danvers, MA, USA), FLAG (1:2000, ab205606, RRID: AB_2916341; Abcam, UK), LAMIN B (1:2000, ab16048, RRID:AB_443298; Abcam) and HRP anti-rabbit secondary (1:10,000, A16110, RRID:AB_2534782; Thermo Fisher Scientific), diluted in blocking buffer [3% (w/v) bovine serum albumin dissolved in tris-buffered saline containing 0.1% (v/v) Tween 20]. Clarity western ECL substrate (Bio-Rad) was used to develop the blots and imaged using the ChemiDoc Touch Imaging system (Bio-Rad).

### Embryo collection and processing

Wild-type, *Zic3^ins5V^*, *Zic3^LacZ^* and *Zic3^V5^* embryos were produced by crossing heterozygous females with either wild-type or hemizygous males and collected at 7.5 to 14.5 days post coitum (dpc). Embryos were fixed in paraformaldehyde (for histology or *in situ* hybridization) or processed for RT-PCR as described previously (Bellchambers and Ware, 2021). Organ arrangement and heart looping phenotypes were assessed before the hearts were paraffin-embedded, sectioned and stained with Hematoxylin and Eosin as described elsewhere (Bellchambers and Ware, 2021; Haaning et al., 2013; Ware et al., 2006a).

### Quantitative RT-PCR

Quantitative RT-PCR was performed on *n*=3 wild-type and *n*=5 *Zic3^ins5V^* embryos at the headfold stage (7.75 dpc). RNA was isolated using the cells-to-Ct kit (Thermo Fisher Scientific, Waltham, MA, USA), reverse transcribed to cDNA and analyzed via RT-PCR as described before (Bellchambers and Ware, 2021) using the 7500 real-time PCR system (Applied Biosystems, Thermo Fisher Scientific) and Taqman probes (FAM-MGB probes: 4448892; VIC-MGB probes: 4448489) for *Zic3* (Mm00494362_m1) and *Tbp* (Mm01277042_m1; for data normalization).

All probes were selected to span exons and therefore not detect gDNA. Three biological replicates were performed for all samples/assays. The reactions were carried out in triplicate for each cDNA sample and their average used to calculate ΔCT values. Relative gene expression was calculated using the 2^−ΔΔCT^ method (Livak and Schmittgen, 2001). The Student's *t*-test (two-tailed) was performed on ΔCT values using Excel 2016 (Microsoft, Redmond, WA, USA). Geometric means and geometric standard deviations (SD) were calculated using GraphPad Prism 10.2.3 (Dotmatics, Boston, MA, USA), which was also used to plot graphs.

### Whole-mount *in situ* hybridization

Paraformaldehyde-fixed wild-type and *Zic3^ins5V^* embryos were dehydrated through a methanol series. Whole-mount *in situ* hybridization was performed as described previously (Purandare et al., 2002; Ware et al., 2006b). RNA probes were generated from plasmid DNA using the DIG RNA labelling kit (Roche, MilliporeSigma). The *Zic3 in situ* probe has been described previously (Purandare et al., 2002).

### Viability analysis

For the viability screen, wild-type males were crossed with heterozygous *Zic3^ins5V/wt^* females. Pups were genotyped at 2 weeks of age or 13.5-14.5 dpc to calculate the Mendelian ratios. Statistical significance was calculated via Chi-Squared test using Excel 2016 (Microsoft).

### Materials availability

All mouse lines and plasmids generated in this study are available from the corresponding author with a completed materials transfer agreement.

## Supplementary Material

10.1242/biolopen.061677_sup1 Supplementary information
