# Supplementary material for: A CRISPR mis-insertion in the Zic3 5′UTR inhibits in vivo translation and is predicted to result in formation of an mRNA stem-loop hairpin
Source: Biol Open. 2025 Mar 17;14(3):bio061677. doi: 10.1242/bio.061677 (PMC11957448; doi:10.1242/bio.061677)
Supplement: Supplementary information [file biolopen-14-061677-s1.pdf]

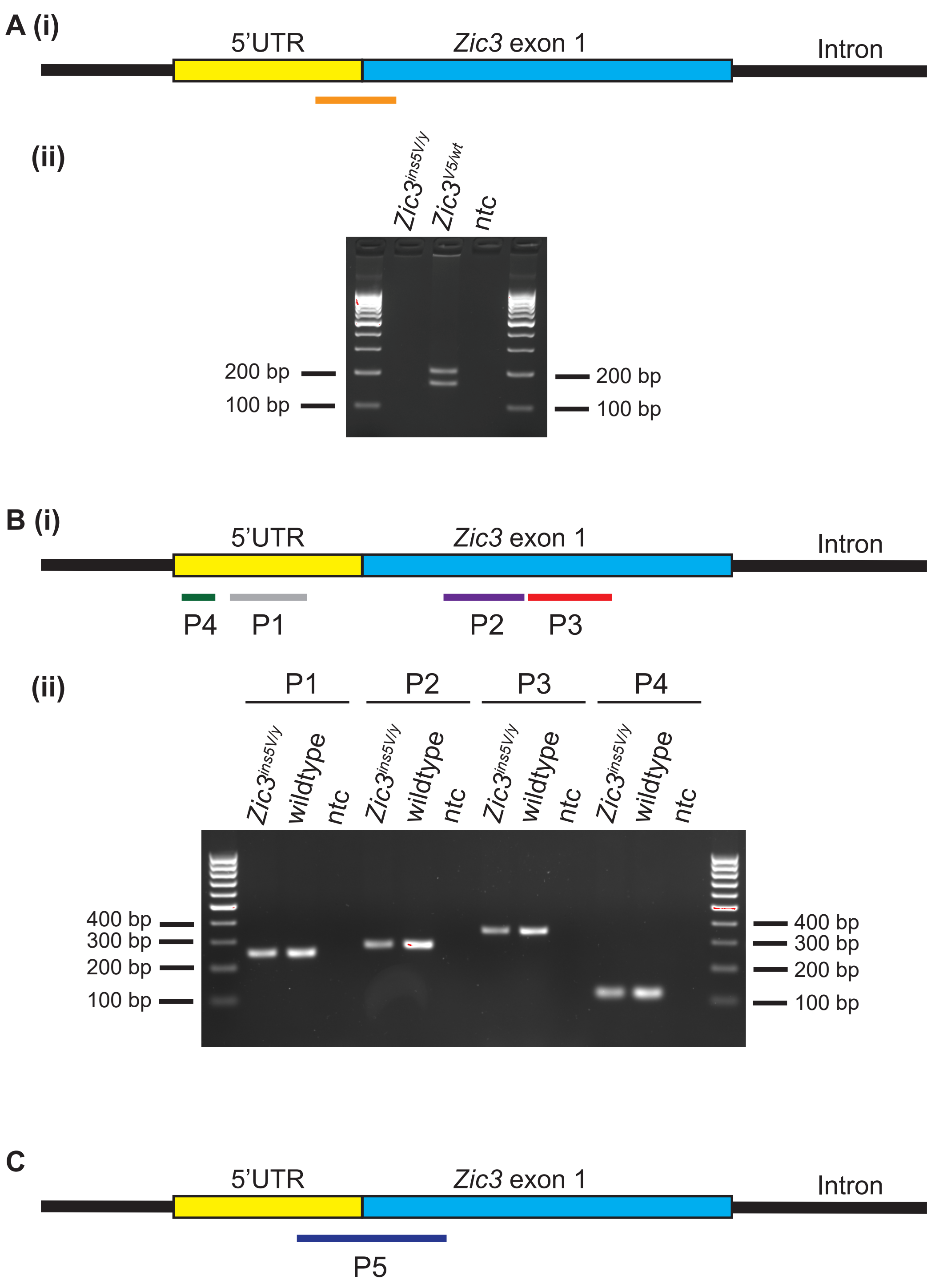

Fig. S1. PCR analysis of *Zic3*^{ins5V}. (A) (i) Schematic illustrating the region targeted by the initial genotyping PCR. (ii) Electrophoretogram showing fragments produced by the PCR in (i). (B) (i) Schematic illustrating the regions amplified by four PCRs of which two target the *Zic3* 5'UTR and two target *Zic3* exon 1. (ii) Electrophoretogram showing fragments produced by the PCRs in (i). P1-P4 indicate the regions targeted by the different primer pairs. (C) Schematic illus-trating the region amplified by the PCR in Fig. 1. The corresponding agarose gel is shown in Fig. 1C. P5 indicates the regions targeted by the PCR.

Biology Open • Supplementary information

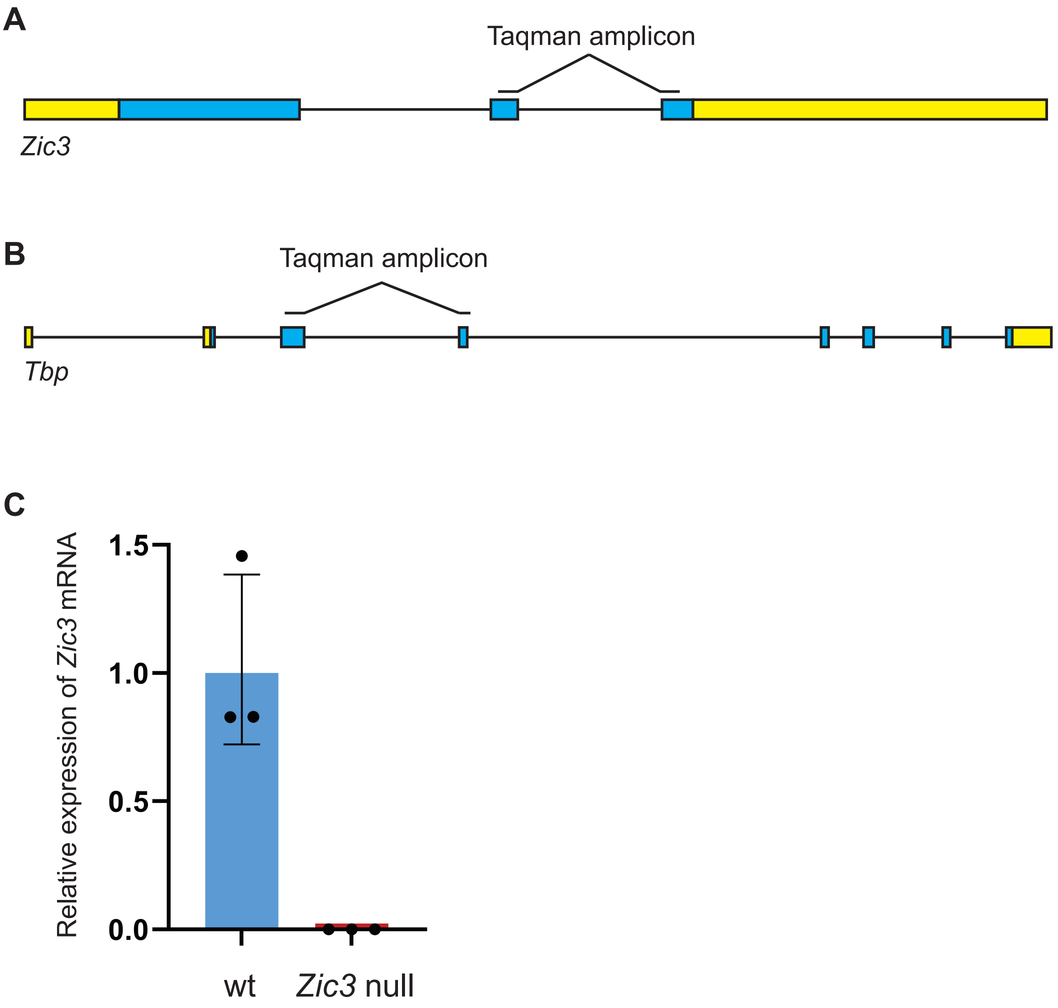

Fig. S2. Target and specificity of the *Zic3* qPCR probe. (A) Schematic illustrating the region targeted by the *Zic3* qPCR amplicon. (B) Schematic illustrating the region targeted by the *Tbp* qPCR amplicon. (C) Relative expression levels of *Zic3* in wildtype (n = 3; wt) and *Zic3* null (n = 3) embryos at the early somite stage (8.25 dpc) assessed using Taqman probes for qPCR shown in A-B. *Tbp* was used for data normalization. Bar graph represent geometric mean and dots represent individual samples. Error bars represent geometric standard deviation (SD).

Biology Open • Supplementary information

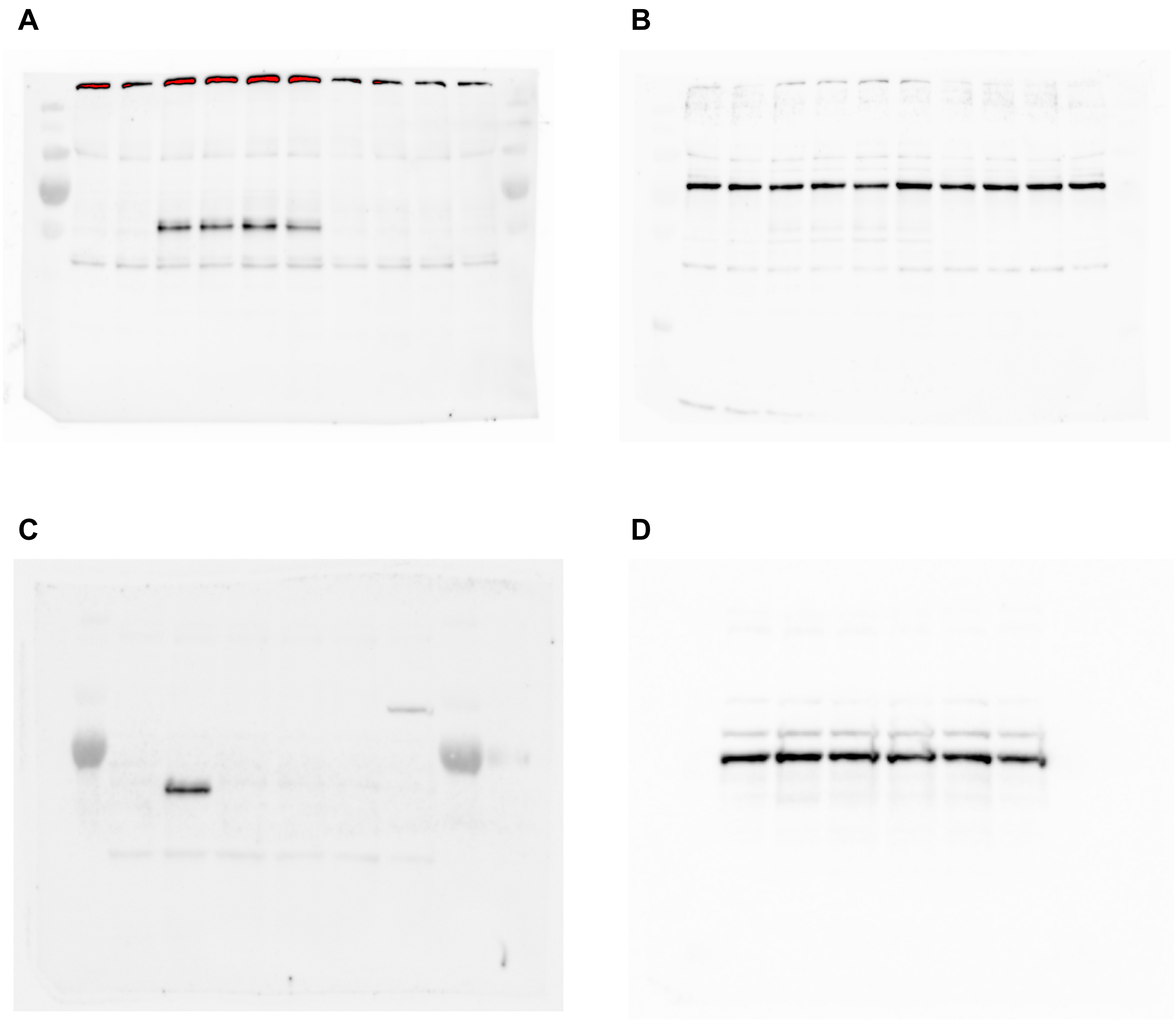

Fig. S3. Uncropped western blots from Figs. 4 and 5. (A-B) Uncropped versions of Fig. 4D western blots for (A) V5 and (B) LAMIN B (nuclear loading control). (C-D) Uncropped versions of Fig. 5D western blots for (A) FLAG and (B) LAMIN B (nuclear loading control).

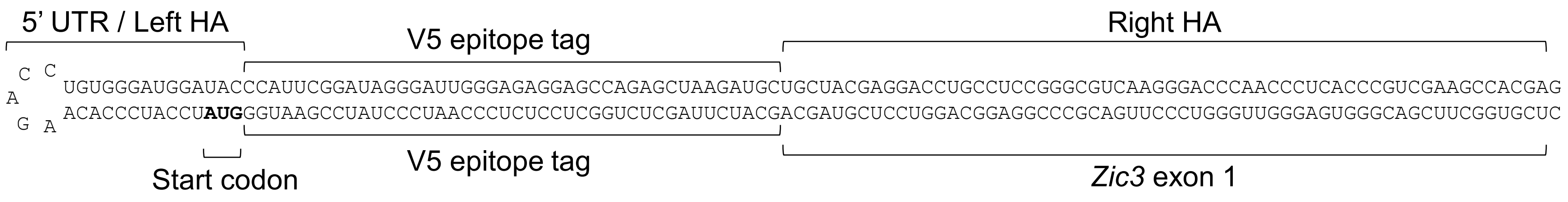

Fig. S4. Full sequence of predicted *Zic3*^{ins5V} mRNA hairpin. HA, homology arm.

Biology Open • Supplementary information

Table S1. Primers used to amplify and/or sequence mouse *Zic3*

Name	Purpose	Sequence
Zic3 P1 F	To amplify and sequence mouse *Zic3* (5'UTR from -380 to -135)	CGCGCTCTTGAGTAGAGGAG
Zic3 P1 R		CCGGAGCTGCAACTTTCCTA
Zic3 P2 F	To amplify and sequence mouse *Zic3* (exon 1 from 225-501)	ACCGCAGGGTTCAGGTTATG
Zic3 P2 R		CTGCTCATGAAGCCCAGGAA
Zic3 P3 F	To amplify and sequence mouse *Zic3* (exon 1) 482-827	TTCCTGGGCTTCATGAGCAG
Zic3 P3 R		TCATGCATGGTGCTGAAGGT
Zic3 P4 F	To amplify and sequence mouse *Zic3* (5'UTR from -522 to -421)	CTGGAGGTGAAGAGGCTTGG
Zic3 P4 R		GAGCAACTGCACCAAGAAGC
Zic3 P5 F	To amplify and sequence mouse *Zic3* across the insertion (from -166 to +243)	AGACTCTCGCAGCCTAGGAA
Zic3 P5 R		ATAACCTGAACCCTGCGGTG
mZic3 exon 1 F2	To amplify and sequence mouse *Zic3* (exon 1) 711-1057	CTTCTTCCGTTACATGCGGC
mZic3 exon 1 R1		TCTCTGGCTGTTCAGTCTGC
mZic3 exon 2 F	To amplify and sequence exon 2 of mouse *Zic3*	TTCAATCTCCCGCTCACCAC
mZic3 exon 2 R		TGTTGGAATCAGACTGCGCT
mZic3 exon 3 F	To amplify and sequence exon 3 of mouse *Zic3*	CCCAATCCATCCACGGAGAA
mZic3 exon 3 R		TCCAGAACGGGGTGTACAGA

Biology Open • Supplementary information
